# Making a diagnosis of hypertension and defining treatment threshold in very low birth weight infants’ need revision?

**DOI:** 10.15171/jrip.2016.13

**Published:** 2016-06-01

**Authors:** Sreekanth Viswanathan, Deepak Kumar, Craig Sykes, Stephanie Olbrych, Nishant Patel, Dennis M. Super, Jessica Darusz, Rupesh Raina

**Affiliations:** ^1^Rainbow Babies and Children Hospital, Cleveland, Ohio, USA; ^2^MetroHealth Medical Center, Cleveland, Ohio, USA; ^3^Akron Children Hospital, Akron, Ohio, USA

**Keywords:** Hypertension, Very low birth weight infants, Blood pressure, Prematurity

## Abstract

**Introduction:** Recent evidence suggests that preterm birth is a possible risk factor for high blood pressure (BP) in later life. The most widely quoted blood pressure centiles for very low birth weight (VLBW, ≤1500 g birth weight) infants at corrected term gestation is based on a cohort with mostly late preterm or term infants (Zubrow curves).

**Objectives:** The objective of this study was to determine the clinical utility of the Zubrow curves in diagnosis of hypertension in VLBW infants at their term corrected gestational age (CGA).

**Patients and Methods:** In a case-control study, we compared BP in 75 VLBW infants at 40 weeks CGA (cases) to 69 full term infants admitted to neonatal intensive care unit (NICU) (controls).

**Results:** In spite of having lower weights, VLBW infants compared to term infants (2612.8 ± 546 vs. 3308.2 ± 373 g, *P* ≤ 0.001) had higher average systolic (88.8 ± 7.6 vs. 82.33 ± 8.5 mm Hg; *P* ≤ 0.001) and mean BP (61.2 ± 6.6 vs. 57.61 ± 6.9, *P* = 0.01). Although 41% (31/75) VLBW infants would have met the criteria for hypertension according to Zubrow curves only 4% (3/75) were diagnosed with hypertension.

**Conclusion:** Since Zubrow BP centiles were based on a heterogeneous population of infants including preterm and term infants, new BP centiles based on chronological data from VLBW infants would allow a better definition of hypertension in these infants and identify the threshold BP for initiating treatment.

**Implication for health policy/practice/research/medical education:**

The exact incidence of hypertension in very low birth weight (VLBW) infants at corrected term gestation is difficult to estimate due to lack of a robust and universally accepted definition for hypertension. Current blood pressure (BP) nomograms for VLBW infants are based on a limited number of VLBW infants followed to term corrected gestational age (CGA). There is a need for a large multicenter study to develop more appropriate gestational age or birth weight based BP centiles with chronological data from birth through term corrected gestation age, infancy and beyond.

## Introduction

There is emerging evidence that preterm birth is associated with increased risk for hypertension later in life (1-4). A large national cohort study in Sweden found that young adults who were born preterm have higher odds of hypertension diagnosis compared to full term infants (1.25 [CI: 1.12-1.39]) (2). The odds of hypertension diagnosis were even higher for those born extremely preterm (2.51 [CI: 1.11-5.68]) (2). In another large cohort of 18 year-olds, the risk of elevated systolic blood pressure (SBP) was twofold higher in premature infants born between 24-28 weeks gestational age (5). In a study of middle-aged individuals born preterm, SBP reduced by 0.53 mm Hg for every one week increases in gestational age at birth (6). With increased survival of very low birth weight (VLBW) infants, the population of ex-premature infants at risk for developing hypertension and cardiovascular morbidities in future is increasing.

VLBW infants, compared to term infants, are more frequently exposed to multiple risk factors (maternal hypertension, umbilical arterial lines, steroids, acute kidney injury) during their prolonged neonatal intensive care unit (NICU) stay and are at risk for developing chronic lung disease (CLD-need of oxygen at 36 week corrected gestational age [CGA]), which can potentially elevate their BP (7). The most widely used post conceptional ag**e** BP nomogram, for infants admitted to NICU, was derived from a cohort, mostly consisting of late preterm or term infants (Zubrow curves, Philadelphia Blood Pressure Study Group1995) (8). From our experience, BP in a significant proportion of VLBW infants at term CGA would meet the definition of hypertension based on Zubrow curves (above the 95th percentile of systolic BP). Since the reported incidence of hypertension ranges from 0.2% to 3% (7), we speculate that neonatologists are often comfortable to diagnose hypertension in these VLBW infants at higher BP thresholds than that are suggested by Zubrow curves.

## Objectives

The objective of this study was to determine the clinical utility of the Zubrow curves in diagnosis of hypertension in VLBW infants at their term CGA.

## Patients and Methods

### Study population

MetroHealth NICU is a level III unit with 50 beds serving a diverse population from Cleveland and North East Ohio with over 600 admissions per year including approximately 100-150 VLBW infants/year. Majority of NICU admissions are inborn. This is a retrospective case control study of all VLBW infants admitted to the NICU between March 2009 and May 2011. Cases were defined as VLBW infants who were required to stay in the NICU at CGA of 40 weeks. Controls were defined as term infants of 37-40 weeks of gestation who required admission to the NICU and remained in the NICU at the CGA of 40 weeks. For comparison, the controls were selected in a manner similar to the term infants included in the Zubrow’s study cohort (Term infants admitted to NICU constituted 1/3 of their total cohort) (8).BP data for term controls was collected only after the first five days of life to avoid including the immediate postnatal BP changes (8). Patients with significant anomalies including cardiac and renal anomalies were excluded. Medical records were reviewed for infant characteristics. Infants in both cases and controls were categorized as stable or unstable. Infants were considered unstable if they were requiring respiratory support (noninvasive or mechanical ventilation), or treatment with inotropes, diuretics, steroids, narcotics or sedative medications at 40 weeks CGA. Average weekly systolic (SBP), diastolic (DBP) and mean blood pressure (MBP) at 40 weeks CGA were was calculated for both groups. BP was measured as per the unit protocol, using an automated oscillometric device with the appropriate cuff placed on the upper arms or calves of the leg (avoiding the extremity with intravenous access) when infants were lying supine or prone, in a calm state or asleep. The unit protocol recommends at least one BP measurement per 8 hour shift in stable growing VLBW infants and more frequent BP measurement in unstable infants. Any BP reading felt to be abnormal is confirmed by repeating two to three times. The BP values documented in the electronic medical records were abstracted and the average BP at 40 week CGA for each infant was calculated. BP and infant characteristics between cases vs. controls were compared at CGA of 40 weeks.

### Ethical issues

The study protocol was in accordance with the Declaration of Helsinki and was approved by the Ethics Committee of MetroHealth Medical Center, Cleveland, Ohio, United States.

### Statistical analysis

Two-way analysis of variance (ANOVA) (9) was performed using BP at 40 weeks as dependent variable and gestational age (preterm, term) and stability (stable, unstable) as independent variables. A two tailed *P* value <0.05 was considered statistically significant. The statistical software IBM SPSS Statistics version 19 (SPSS, Chicago, IL) was used for the statistical analysis of the data.

## Results

From consecutive 200 eligible VLBW and term infant cohorts admitted during the study period, 75 VLBW infants (cases) and 69 term infants (controls) met the inclusion criteria. Remainder of the infants was either excluded, discharged or had expired before reaching the CGA of 40 weeks.

### Clinical characteristics of cases versus controls

VLBW infants (birth weight 875.1 ± 255 g; gestation 26.9 ± 2.8 weeks.), compared to term infants (3181.3 ± 376 g; 39.1 ± 1.2 weeks.), were more likely to be small for gestational age (24.0 vs. 5.8%, *P*=0.002), African American (61 vs. 40 %, *P*=0.02), have lower body weight at 40 weeks CGA (2612.8 ± 546 vs. 3308.2 ± 373 g, *P*≤0.001), higher exposure to central line days (38.3 ± 37 vs. 7.6 ± 20.2, *P*≤0.001), urinary tract infection (15.9 vs. 0.0%, *P*=0.005) and culture positive bacteremia (48 vs. 13%, *P*≤0.001). There was no significant difference in exposure to maternal preeclampsia, postnatal steroids and acute kidney injury between VLBW infants and term infants. 27/75 (36.0%) of the VLBW infants and 12/69 (17.4%) of the term infants were categorized as unstable (*P*=0.15). The incidence of CLD in VLBW infants admitted during the study period was 33% (66/200), but the incidence was higher in the selected case group of VLBW infants who were required to stay in the NICU at 40 weeks CGA (63% [47/75]).

### Blood pressure in cases versus controls

**Figure 1:**
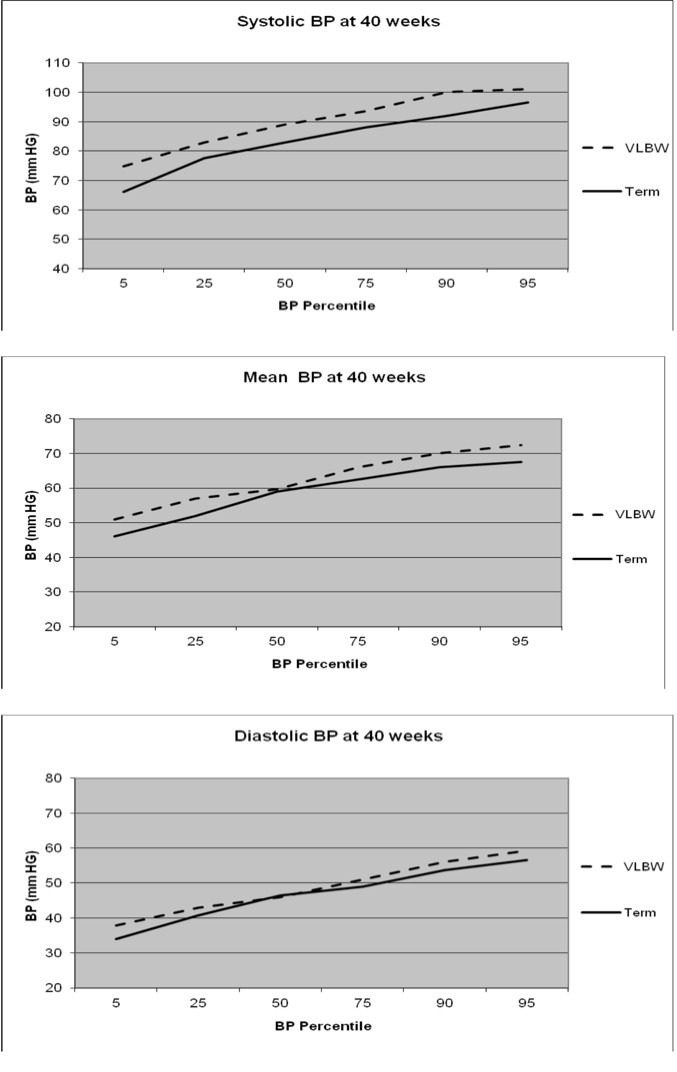
Percentile curves of SBP, MBP and DBP of VLBW infants and term infants at the corrected gestation on of 40 weeks.

**Figure 2:**
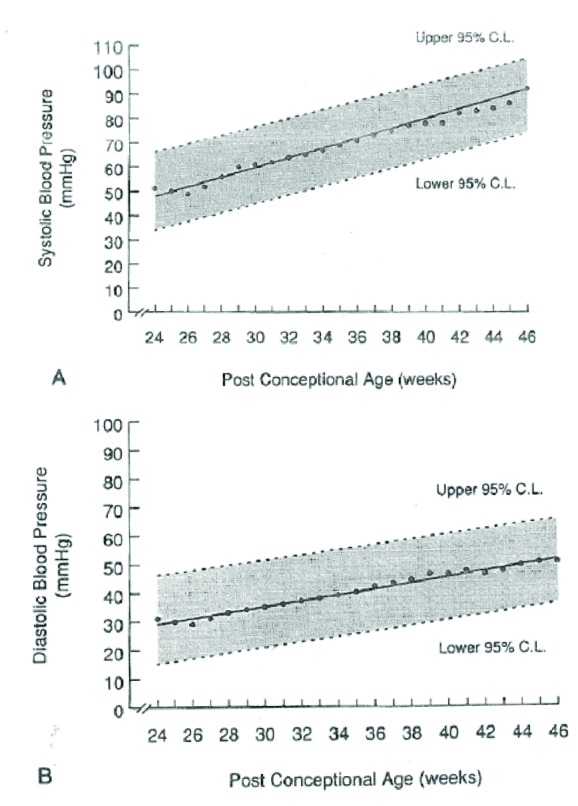
Linear regression of mean systolic (A) and diastolic (B) blood pressures (BPs) by postconceptional age in weeks, with 95% confidence limits. Reproduced from Zubrow et al (8) with permission from the Nature Publishing Group.

Cases compared to controls had higher mean SBP (88.8 ± 7.6 vs. 82.33 ± 8.5 mm Hg; *P*≤0.001) and mean MBP (61.2 ± 6.6 vs. 57.61 ± 6.9, *P*=0.01), but similar DBP (47.38 ± 6.5 vs. 45.25 ± 6.5, *P*=0.15) (Figure 1). The results were unchanged after adjusting for the main effect of infant stability (stable or unstable) and there was no significant interaction between stability and gestational age (preterm, term) in the two way ANOVA**.** The 95th percentile values for cases vs. control infants were: SBP (101.0 vs. 96.5 mm Hg), MAP (72.40 versus 67.5), and DBP (59.2 vs. 56.5) (Figure 1). The 95th percentile for SBP at 40 weeks based on Zubrow curves;graphically corresponds to 90 mm Hg (Figure 2) (8). In our study, 41% (31/75) of VLBW infants at CGA of 40 weeks would have met the definition of hypertension based on Zubrow curves, but only 4%(3/75) of infants were diagnosed with hypertension before discharge from the NICU.

### VLBW infants with hypertension

In VLBW infants who were diagnosed with hypertension, the average SBP at CGA of 40 weeks was 100 mm Hg. Amongst the 75 VLBW infants, infants with CLD (47/75) compared to infants without CLD (28/75) had lower birth weight (784.5 ± 197.5 vs. 1053.2 ± 238.3 g, *P*≤0.001), lower gestational age at birth (25.8 ± 2.2 vs. 28.8 ± 2.5 weeks, *P*≤0.001), but had similar weight (2561.1 ± 516.5 versus 2783.3 ± 488.7 g, *P*=0.07) and BP (Mean SBP [88.3 ± 8.1 vs. 89.8 ± 6.9, *P*=0.40], MAP [60.9 ± 7.0 vs. 61.6 ± 6.2, *P*=0.65], DBP [47.2 ± 6.3 vs. 47.6 ± 6.9, *P*=0.78]) at 40 weeks CGA.

## Discussion

The natural history of BP in preterm infants, particularly in VLBW infants, is not as well described as in term infants. Most studies describing normative BP pattern in premature infants have focused on the immediate postnatal period (10). Zubrow BP curves are the most widely quoted BP percentile curves for premature infants 24-46 weeks CGA and were derived from a prospective study that collected serial BP measurements from both term and preterm infants admitted to several NICUs (8). But, our data shows that VLBW infants at 40 weeks CGA have higher BP compared to full term infants admitted to NICU. More than a third of our VLBW infants at 40 weeks CGA could potentially fit the criteria for hypertension per Zubrow curves but only 4% were diagnosed with hypertension. Utility of Zubrow curves to determine the optimal BP threshold for the diagnosis and treatment of hypertension in VLBW at their term CGA is uncertain.

The reported incidence of hypertension in neonates is relatively low and ranges from 0.2%-3% (7). In a review of over 3000 infants admitted to a NICU, the overall incidence of hypertension was 0.81%, but it is as high as 9% in high risk premature infants with CLD, patent ductus arteriosus, intraventricular hemorrhage or in those that had indwelling umbilical arterial catheters (11). Not all cases of hypertension in premature infants are detected while in the NICU. In a retrospective study of over 650 preterm infants (<37 weeks gestation) discharged from the NICU, the incidence of hypertension was 2.6% over 6-42 month follow up period and average time to diagnosis hypertension was at two months CGA(12). In their study, the infants who developed hypertension had a longer NICU stay compared to normotensive infants (38.8 ± 10.2 vs. 29.1 ± 4.7 days) suggesting higher likelihood of developing hypertension in sicker infants (12).

When dealing with a high BP value in an otherwise asymptomatic infant, neonatologists often observe the BP trends over a period of time, while trying to minimize the environmental effects on the BP measurement. Hypertension in neonates is usually diagnosed when the SBP is >95th percentile for age. Feld and Corey have suggested treatment for hypertension if SBP is either > 99th percentile or >95th percentile with end-organs involvement (13).

To guide the optimal management of hypertension in VLBW infants at term CGA, there is no specific BP percentile curves based exclusively on chronological data from a stable VLBW cohort. Zubrow BP curves (24-46 weeks CGA percentiles) were developed in 1994 based on a prospective, multicenter study of 608 neonates admitted to 14 different NICUs in Philadelphia over the first 99 days of life (8). But VLBW infants constituted only one fifth of this cohort. Further, the BP values of VLBW infants were chronologically reported as pooled values along with bigger and more gestationally mature infants, and included infants on ventilator and inotropic support. Several studies have shown that BP in premature infants shows a rapid increase over the first 1-2 weeks followed by a gradual increase with increasing chronological age (7,8,10). In Zubrow’s study, the inclusion of BP data from preterm infants during the first 2 weeks of life when rapid changes in BP dynamics occur could have also confounded the data. In their regression analysis, only the post conceptual age was the primary determinant of BP but not the birth weight or gestational age at birth. Based on these curves, infant SBP is considered to be elevated if it falls above the 95th percentile of the mean SBP for infants of similar CGA irrespective of the birth gestation. Similarly Kent et al studied the BP data of non-ventilated stable premature infants (28-36 weeks gestation) in their first month of life, and concluded that premature neonates stabilize their BP after 14 days of life and after this their BP is similar to that of term infants (14). Based on Zubrow curves, 41% of VLBW infants at 40 weeks CGA in our study would fit the diagnosis of hypertension. It is plausible that VLBW infants at their term CGA indeed have higher baseline BP compared to term infants, so pooling them together as in Zubrow’s study may result in lower BP percentiles. Also the low rate of hypertension diagnosis even in these high-risk VLBW infants suggests the neonatologist’s tolerance for higher BP threshold for diagnosing hypertension than suggested by Zubrow curves.

Dionne et al have constructed a clinically useful reference table of estimated BP values (50th, 95th, and 99th percentile) after the first 2 weeks of age in infants from 26 to 44 weeks CGA based on previous published studies (7,8,10,15-19). Of the seven studies from which data was collated for generating these reference values, only three studies included VLBW infants – Zubrow et al (8), Kent et al (10), Pejovic et al (16). Both Kent et al and Pejovic et al studies reported BP values in the first month of life only. Zubrow’s study is the only published study that reported the BP of VLBW infants up to their term CGA. Based on the Dionne and Flynn reference table, at 40 weeks CGA, the 95thpercentile for SBP is 95 mm Hg, five points higher than Zubrow curves. And the 99th percentile at 40 weeks CGA corresponds to 99 mm Hg, which is very similar to the BP values at which infants were diagnosed with hypertension in our study (average SBP of 100 mm Hg). Though this reference table may reflect values similar to the current clinical practice, it is still a concern that it is based on a limited number of VLBW infants that were followed to term CGA.

The organs that develop late in-utero, such as kidneys, lungs or eyes, are most at risk of altered remodeling and development in the prematurely born sick newborn. Nephrogenesis is completed by 34-35 weeks gestation (14). Final nephron endowment depends on both genetic and environmental factors (9) whereby adverse perinatal events may modify the nephrogenesis and resulting nephron under dosing (‘low nephron number hypothesis’), causing the remaining nephrons to undergo hypertrophy and hyper filtration in order to sustain renal function, which results in glomerular injury and an elevated BP (20,21). The extent of postnatal renal growth and nephrogenesis in VLBW infants may not be optimal leading to compensatory hyper filtration in some infants (9).Animal studies has shown that protein restriction of the mother in the last trimester (the most intense period of nephrogenesis) results in 20%-30% reduction in nephron number and subsequent development of hypertension (22). Nephrogenesis and renal vascularization are intimately related (14). Experimental evidence suggests that during periods of stress, there is decreased vascular arborization in the fetus or neonate leading to microvascular rarefaction (reduced density of arterioles and capillaries) which is associated with hypertension (14). This has been suggested as the primary event in the development of hypertension associated with early fetal stress. Some increase in BP thus may be an adaptive mechanism to maintain adequate tissue perfusion (23,24). Elastin synthesis in blood vessels peak during the third trimester and then decreases rapidly after birth (14,24). Prematurity and low birth weight are associated with increased arterial stiffness later in life due to increase in collagen content relative to elastin (24). Prematurity related early neonatal stress may be associated with vascular oxidative injury and endothelial dysfunction causing abnormal central and muscular vascular remodeling (9,14).Thus hypertension in children born prematurely is likely multifactorial including abnormal nephrogenesis and vascularization, growth restriction, increased susceptibility to acute kidney injury and exposure to nephrotoxic drugs.

The VLBW infants in our cohort were sick enough to continue in the NICU at 40 weeks CGA and a significant number had CLD. CLD in premature infants has been previously reported to be associated with higher incidence of hypertension and more than half of these were diagnosed with hypertension after NICU discharge (7,12,25). Concurrent nephrocalcinosis in these premature infants is a significant risk factor for developing late onset hypertension (26). VLBW infants without CLD still admitted to the NICU at 40 weeks CGA in our study had similar elevated BP as infants with CLD, suggesting that being born as VLBW infant and exposure to various risk factors during the NICU stay could probably lead to the higher BP. The National High Blood Pressure Education Program Working Group on high blood pressure in children and adolescents, in their fourth report, recommended routine BP monitoring in all children after three years of age but even earlier in premature, VLBW or sick infants requiring initial NICU stay (27). Practically, it is uncertain how widely are these recommendations being followed.

## Conclusion

In spite of having lower body weights, VLBW infants at 40 weeks corrected gestation, have higher systolic and mean BP compared to term infants, which exposes them to an elevated risk for cardiovascular morbidities later in life. Preterm infants especially VLBW infants are at higher risk of developing hypertension as a result of early life postnatal physiological programming, deficits in nephron endowment and exposure to adverse environmental events during their NICU stay. Currently available BP nomograms for VLBW infants are based on a limited number of VLBW infants followed to term CGA. The exact incidence of hypertension in VLBW infants at corrected term gestation is difficult to estimate due to lack of a robust and universally accepted definition for hypertension. There is a need for a large multicenter study to develop more appropriate gestational age or birth weight based BP centiles with chronological data from birth through term corrected gestation age, infancy and beyond. VLBW infants should be monitored for elevated BP and altered renal function throughout infancy, childhood and adulthood.

## Limitations of the study

Our study has several limitations. It is retrospective case control study from a single center NICU. Although, we have a written NICU protocol for BP monitoring, BP documented in the chart may be have been affected by infant temperament, feeding and infant position. Although our protocol requires it and frequently the nursing documentation comments upon these factors, it is conceivable that documentation may not have been 100% compliant. BP measurement using oscillometric manometers may be less accurate than the gold standard intra-arterial monitors but studies have shown good correlation between oscillometric and umbilical artery BP in neonates (12). Though upper limb BP is traditionally considered as standard, we included calf BP as well. Published data suggests a wide and random variation in reported BP readings between upper limbs and lower limbs in newborn infants (13,15). These studies also reported minimal or no difference between BP recordings from arm or calf perhaps due to small muscle mass in these infants.

## Acknowledgments

This study was made possible by the Edward M. Chester MD Summer Scholars Program at Metro Health Medical Center.

## Authors’ contribution

All the authors have contributed towards performing the study and preparation of the manuscript and they all have approved the latest version of the article. SV and DK conceptualized the study, participated in data collection, analysis-interpretation, manuscript writing and editing. CS, SO, and NP assisted in data collection, analysis, manuscript writing and editing. DMS helped finalize the methodology, data analysis and interpretation and manuscript editing. JD helped with compiling document elements for submission and manuscript editing. RR helped with data analysis and interpretation, manuscript preparation and editing.

## Funding/Support

The authors have no financial relationships relevant to this article to disclose.

## Ethical considerations

Ethical issues (including plagiarism, data fabrication, double publication) have been completely observed by the authors.

## Conflicts of Interest

The authors have no conflicts of interest to disclose.
